# Evaluation of a strategy using pretherapeutic fiducial marker placement to avoid missing liver metastases

**DOI:** 10.1002/bjs5.50140

**Published:** 2019-02-22

**Authors:** V. Kepenekian, A. Muller, P. J. Valette, P. Rousset, M. Chauvenet, G. Phelip, T. Walter, M. Adham, O. Glehen, G. Passot

**Affiliations:** ^1^ Department of Digestive Surgery Hospices Civils de Lyon, Centre Hospitalier Lyon Sud Lyon France; ^2^ Department of Radiology Hospices Civils de Lyon, Centre Hospitalier Lyon Sud Lyon France; ^3^ Department of Digestive Oncology Hospices Civils de Lyon, Centre Hospitalier Lyon Sud Lyon France; ^4^ Department of Medical Oncology Hospices Civils de Lyon, Hôpital Edouard Herriot, Lyon 1 University Lyon France; ^5^ Department of Digestive Surgery Hospices Civils de Lyon, Hôpital Edouard Herriot, Lyon 1 University Lyon France

## Abstract

**Background:**

Hepatic surgery is appropriate for selected patients with colorectal liver metastases (CRLM). Advances in chemotherapy have led to modification of management, particularly when metastases disappear. Treatment should address all initial CRLM sites based on pretherapeutic cross‐sectional imaging. This study aimed to evaluate pretherapeutic fiducial marker placement to optimize CRLM treatment.

**Methods:**

This pilot investigation included patients with CRLM who were considered for potentially curative treatment between 2009 and 2016. According to a multidisciplinary team decision, lesions smaller than 25 mm in diameter that were more than 10 mm deep in the hepatic parenchyma and located outside the field of a planned resection were marked. Complication rates and clinicopathological data were analysed.

**Results:**

Some 76 metastases were marked in 43 patients among 217 patients with CRLM treated with curative intent. Of these, 23 marked CRLM (30 per cent), with a mean(s.d.) size of 11·0(3·4) mm, disappeared with preoperative chemotherapy. There were four complications associated with marking: two intrahepatic haematomas, one fiducial migration and one misplacement. After a median follow‐up of 47·7 (range 18·1–144·9) months, no needle‐track seeding was noted. Of four disappearing CRLM that were marked and resected, two presented with persistent active disease. Other missing lesions were treated with thermoablation.

**Conclusion:**

Pretherapeutic fiducial marker placement appears useful for the curative management of CRLM.

## Introduction

Colorectal liver metastases (CRLM) occur in about 60 per cent of patients with colorectal cancer^1^. Surgery, often in association with thermoablation^2^, offers these patients the best chance of prolonged survival^3^, ^4^, with 5‐year survival rates of over 50 per cent and a curative rate of 17 per cent^4^. However, only 25 per cent of CRLM are resectable^5^, ^6^ at the time of diagnosis, although up to 20 per cent of CRLM initially assessed as unresectable become resectable after chemotherapy^7^, ^8^, ^9^.

Preoperative chemotherapy is the treatment of choice in patients with borderline resectable or unresectable CRLM, especially when the carcinoembryonic antigen level is raised and performance status is preserved^10^, ^11^, ^12^. It has been estimated^13^, ^14^ that in 9–37 per cent of patients treated with preoperative chemotherapy at least one CRLM will disappear after preoperative chemotherapy, challenging the curative intent strategy. Despite a complete radiological response, a complete pathological response is estimated to occur in anything from 17 to 80 per cent of these patients^13^, ^14^, ^15^, ^16^, ^17^, ^18^, ^19^, ^20^, ^21^, ^22^, ^23^, ^24^. A previous study described two types of vanishing lesion: disappearing lesions, defined as CRLM that disappear from the future resected liver; and missing metastases (MM), defined as CRLM that disappear but remain part of the future liver remnant^15^.

According to a recent consensus statement^16^, the goal of hepatic resection should be to remove all original sites of disease with a technique of maximum parenchymal sparing. The efficacy of this approach is jeopardized by MM^17^, ^18^. Marking those lesions at risk of disappearing using a fiducial marker was proposed to facilitate the elective treatment of these areas^25^. This pilot investigation was aimed to evaluate this strategy, focusing on marking‐related complications.

## Methods

From August 2009 to December 2016, all patients who presented with pathologically proven potentially curative CRLM and who were considered for preoperative chemotherapy at the tertiary University Hospital of Lyon (Hospices Civils de Lyon) were registered in the study. Approximately 100 patients with CRLM are treated there each year. This pilot investigation was performed according to the Declaration of Helsinki. Patients signed a dedicated informed consent before the marking during a preinterventional consultation (ethical considerations are detailed in *Appendix S1*, supporting information).

### Patient selection

**Figure 1:**
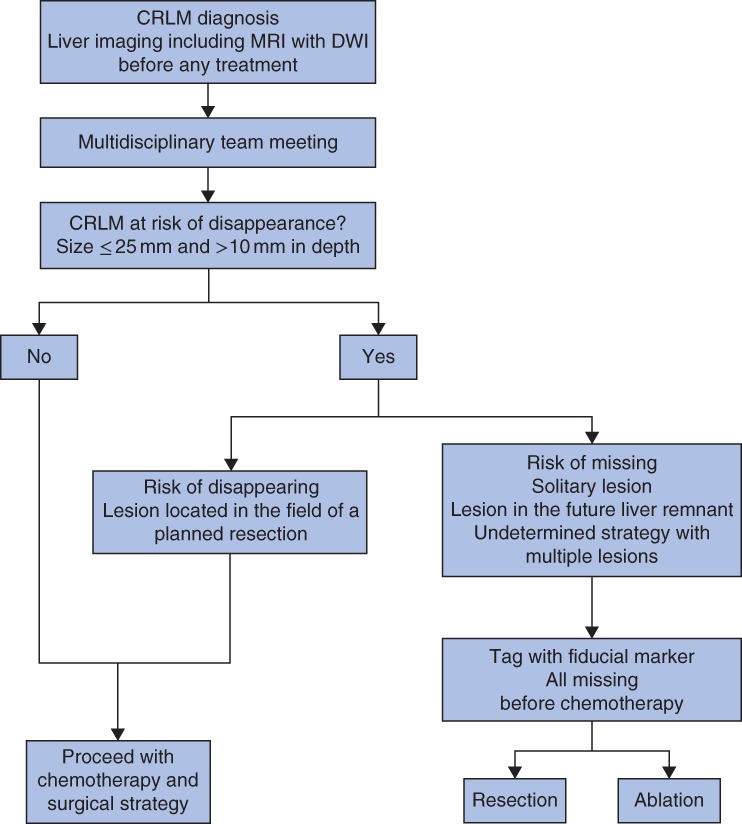
Strategy for fiducial marker placement for lesions at risk of disappearance. CRLM, colorectal liver metastases; DWI, diffusion‐weighted imaging

An initial comprehensive radiological assessment was performed in all patients and ideally included at least a body scan by enhanced multidetector CT (MDCT), along with gadolinium‐enhanced MRI with diffusion‐weighted imaging (DWI) (*Fig*. 1). Before starting systemic chemotherapy, each patient was evaluated in a multidisciplinary team (MDT) meeting that included hepatobiliary surgeons, interventional radiologists and medical oncologists. After reassessment of cross‐sectional imaging, the therapeutic strategy was defined and potentially resectable patients were then determined. All liver lesions that remained uncertain after imaging, and thus not deemed to be assessed as CRLM, were biopsied using contrast‐enhanced ultrasound imaging or CT guidance. CRLM were deemed resectable when a hepatectomy could achieve a negative margin while preserving more than 30 per cent of the total estimated liver volume, sparing two continuous liver segments, and maintaining vascular inflow/outflow and biliary drainage^20^, ^26^. Thermoablation was considered as an alternative to surgery for deep lesions (more than 1 cm into the liver capsule) smaller than 2 cm.

Patients received preoperative chemotherapy according to French guidelines, and the regimen was determined during an MDT meeting^27^. The response to chemotherapy was evaluated radiologically with MDCT and/or MRI, every four cycles, according to the Response Evaluation Criteria in Solid Tumours^28^, as well as morphologically^29^. Lesions at risk of disappearance were defined as lesions smaller than 25 mm and more than 10 mm deep in the liver parenchyma.

Metastases at risk were classified into two groups: ‘disappearing’, when the lesion was included in the field of the planned hepatectomy; and ‘missing’, when the lesion was in the future liver remnant or a potential resection would compromise a large amount of the normal liver. When lesions at risk of becoming missing were assigned to ablative treatment, patients were assigned to radiological marking to evaluate the response to chemotherapy.

### Marking technique

Procedures were performed with local anaesthetic (lidocaine 1 per cent) or conscious sedation when more than one lesion required marking. After checking the coagulation profile, a 3‐mm ring precharged in a 137‐mm 18‐G puncture needle (O‐Twist‐Marker®; BIP Medical, Türkenfeld, Germany) was used. Using ultrasonographic guidance, the tip of the needle was placed to deliver the clip at the margin of the lesion, usually at the posterior edge, rather than inside the lesion, to minimize the risk of tumour seeding. In each case, the position of the clip relative to the lesion was described precisely. If lesions could not be visualized by conventional ultrasound imaging, contrast‐enhanced ultrasonography (SonoVue®; Bracco International, Amsterdam, the Netherlands), CT guidance, or the real‐time fusion of ultrasonography and MRI (diffusion weighted or 3D T1 Gd‐enhanced sequences) images were used to localize the metastases. CT and liver MRI were performed before the operation.

All complications following fiducial placements were graded according to the Clavien–Dindo classification^30^, with particular attention to hepatic parenchymal haematoma, fiducial marker migration, needle‐track dissemination and *in situ* recurrence. Fiducial markers located at a distance more than 10 mm from the lesion at the follow‐up imaging were judged to be misplaced.

### Treatment

Surgery was performed under general anaesthesia after abdominal exploration, liver mobilization and intraoperative ultrasonography (IOUS). Each resected specimen was submitted to pathological examination to evaluate the pathological response^5^. Surgery was performed 4–6 weeks after the last chemotherapy session. The goal of surgery was to obtain complete resection of all CRLM with clear margins.

Thermoablation (radiofrequency or microwave ablation) was performed as described previously^2^, either during surgery when associated with liver resection or percutaneously with real‐time ultrasound imaging or CT or fusion imaging guidance, under general anaesthesia.

### Follow‐up

After surgery, all patients were followed up by clinical examination and radiological assessment via MRI or MDCT every 3 months for 2 years, then every 6 months for 3 years, then annually.

### Statistical analysis

Continuous variables are expressed as median (range) or mean(s.d.). The complication rate was assessed by reporting the ratio of the number of complications to the number of procedures. Duration of follow‐up started from the marking placement. Data were analysed according to procedures, patients, marked lesions, disappearing lesions and lesions of less than 5 mm at the time of treatment, considering that the quality of elective treatment would be worse for lesions smaller than this.

## Results

**Figure 2:**
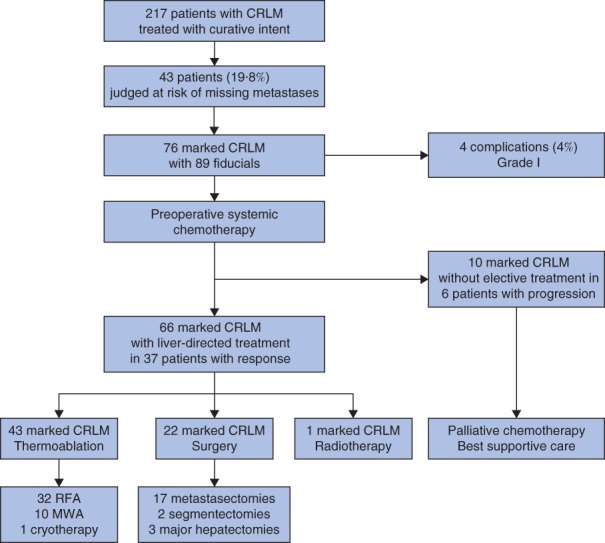
Overview of patient selection and treatment of marked colorectal liver metastases. CRLM, colorectal liver metastases; RFA, radiofrequency ablation; MWA, microwave ablation

Between August 2009 and December 2016, 217 patients with CRLM were treated with curative intent (*Fig*. 2). Among this consecutive group, 43 patients (19·8 per cent) had 76 CRLM that met the criteria for fiducial marking. Patient characteristics are summarized in *Table* 1. Twenty‐one patients (49 per cent) presented with initially unresectable disease. All 43 patients underwent MRI before marking. MRI, MDCT and contrast‐enhanced ultrasound imaging were performed initially in 32 patients (74 per cent), and on the day before liver‐directed treatment in 34 patients (79 per cent). The main drug regimens used were FOLFOX (27 patients), FOLFIRI (10) and FOLFIRINOX (2) with or without a biological agent. The mean(s.d.) number of cycles between the diagnosis and radical treatment was 4·6(1·4) in the 37 patients with no disease progression.

**Table 1:** Patient characteristics

	No. of patients (*n* = 43)
Age at diagnosis (years)*	64·6(8·9)
Sex ratio (M : F)	31 : 12
Site of primary tumour	
Colon	31
Rectum	12
Liver metastases	
No. per patient*	6·6(5·4)
Synchronous	30
Metachronous	13
Solitary	9
Bilobar	30
Initially resectable disease	22
Initially unresectable disease	21
Portal vein embolization	9
Concurrent extrahepatic metastasis	9
Initial imaging evaluation	
MRI + CT + (CE)US + PET	2
MRI + CT + (CE)US	30
MRI + CT	6
CT + (CE)US	5
Preoperative chemotherapy regimen	
FOLFOX	15
FOLFOX + bevacizumab	8
FOLFOX + anti‐EGFR	4
FOLFIRI + bevacizumab	3
FOLFIRI + anti‐EGFR	6
FOLFIRI	1
FOLFIRINOX	2
Capecitabine	4
No. of cycles before hepatic treatment†	4·6(1·4)

Values are *mean(s.d.) and †mean(s.d.) based on 37 patients (6 patients with 14 marked metastases were excluded because of progression). (CE)US, (contrast‐enhanced) ultrasonography; PET, positron emission tomography; EGFR, epidermal growth factor receptor.

Disease progression occurred after marking in six patients (14 per cent) with ten marked metastases. These patients did not undergo the planned hepatic treatment. Of 28 patients who underwent surgery, four developed new CRLM at IOUS not previously identified by preoperative imaging. Fifteen patients had surgery as the sole hepatic treatment, and 21 had a combination of surgery and ablative techniques. In one patient the marked CRLM were treated with radiotherapy. The median duration of follow‐up was 47·7 (range 18·1–144·9) months.

### Marked metastases

Characteristics of the marked metastases are summarized in *Table* 2. Some 76 metastases were marked with 89 fiducial markers. Ultrasound guidance was sufficient to mark 42 CRLM (55 per cent). The mean(s.d.) diameter of the marked lesions was 13·6(5·6) mm at diagnosis and 7·6(7·2) mm at time of specific treatment.

**Table 2:** Characteristics of marked metastases

	No. of marked metastases (*n* = 76)
Size of metastases (mm)	
At diagnosis*	13·6(5·6)
At specific treatment†	7·6(7·2)
Marking techniques	
Total no. of fiducial markers	89
Local anaesthesia (no. of patients)	24
Guiding technique	
US	
No. of CRLM	42
No. of fiducial markers	50
CEUS	
No. of CRLM	13
No. of fiducial markers	15
CT	
No. of CRLM	13
No. of fiducial markers	16
US–MRI	
No. of CRLM	3
No. of fiducial markers	3
US–CT	
No. of CRLM	5
No. of fiducial markers	5
Complications‡	4 (4)
Treatment of marked metastases	
Thermoablation (21 patients)	43 (57)
RFA	32
MWA	10
Cryotherapy	1
Surgery (15 patients)	22 (29)
Metastasectomy	17
Segmentectomy	2
Major hepatectomy	3
Size at diagnosis (mm)*	15·8(6·9)
Size at surgery (mm)*	8·9(8·3)
Histological size (mm)*	11·9(7·1)
Pathological response (% viable cells)*	32(25)
Radiotherapy (1 patient)	1 (1)
No specific treatment (progression) (6 patients)	10 (13)

Values in parentheses are percentages unless indicated otherwise; values are

* mean(s.d.) and

† mean(s.d.) based on 37 patients (6 patients with 14 marked metastases were excluded because of progression).

‡ Total number of complications for total number of fiducial markers placed. CRLM, colorectal liver metastases; US, ultrasonography; CEUS, contrast‐enhanced ultrasonography; RFA, radiofrequency ablation; MWA, microwave ablation.

Of the marked lesions, 43 (57 per cent) were treated using an ablative technique (6 before surgery). Liver resection was performed for 22 of the 76 marked lesions (29 per cent). Parenchymal‐sparing hepatectomy (PSH, metastasectomy) was performed for 17 of these 22 lesions, and five were treated by anatomical resection (2 segmentectomies and 3 major hepatectomies). The mean(s.d.) radiological size of the marked metastases treated by surgery was 15·8(6·9) mm at diagnosis and 8·9(8·3) mm at surgery, with a mean(s.d.) remaining viable cell rate of 32 (25) per cent. At the time of the liver‐directed treatment, 21 of the 43 patients (49 per cent) had 31 marked CRLM smaller than 5 mm. Of these lesions seven (23 per cent) were resected, six using a PSH approach, and ten metastases were not treated because of progression.

### Missing metastases

Twenty‐three of the 76 marked lesions (30 per cent) in 16 patients disappeared (*Table* 3). The mean(s.d.) size of these lesions at diagnosis was 11·0(3·4) mm. All 23 of the MM were treatable: four by surgery, 18 by thermoablation and one by stereotactic radiotherapy. Of the four MM surgically resected, two contained viable residual tumour cells and the two other lesions demonstrated complete histological responses.

**Table 3:** Characteristics of missing metastases and lesions smaller than 5 mm at elective treatment

	No. of marked metastases (*n* = 76)
Missing marked metastases	23 (30)
No. of patients	16
Size at diagnosis (mm)*	11·0(3·4)
Treatment	
Surgery	4
Metastasectomy	3
Major hepatectomy	1
Lesion visible on IOUS	0
Pathological response (% viable cells)	30, 30, 0, 0†
Thermoablation	18
RFA	17
MWA	1
Radiotherapy	1
Marked lesions < 5 mm at elective treatment	31 (41)
No. of patients	21
Size at diagnosis (mm)*	11·5(3·5)
Treatment	
Surgery	7
Metastasectomy	6
Major hepatectomy	1
Thermoablation	23
RFA	21
MWA	1
Cryotherapy	1
Radiotherapy	1

Values in parentheses are percentages unless indicated otherwise;

* values are mean(s.d.).

† Individual pathological responses for the four patients who had surgery. IOUS, intraoperative ultrasonography; RFA, radiofrequency ablation; MWA, microwave ablation.

### Marking‐related complications

**Figure 3:**
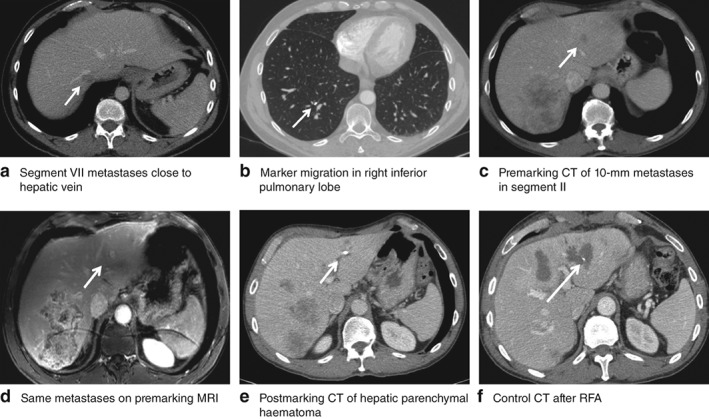
Fiducial marker placement‐related complications. **a,b** Fiducial marker migration: **a** premarking CT scan with metastases of segment VII close to the hepatic vein; **b** postmarking CT scan with fiducial marker migration in the subsegmental branch of the posterobasal segment from the inferior right lobe with no evidence of pulmonary embolism. **c–f** Hepatic parenchymal haematoma: **c** premarking CT and **d** premarking MRI scans showing a 10‐mm metastasis in segment III (white arrow); **e** control CT scan the day after marking showing a hepatic parenchymal haematoma; **f** control CT scan after radiofrequency ablation (RFA)

Four marking‐related complications occurred in 89 procedures: two hepatic parenchymal haematomas, one fiducial marker migration and one fiducial marker misplacement. All complications were grade 1; none required any specific treatment, with no delay in planned chemotherapy. Fiducial marker migration occurred during the marking of a 15‐mm CRLM close to the median hepatic vein. The marker migrated through the hepatic vein to the pulmonary parenchyma, with no clinical consequences (*Fig*. 3 *a,b*). The two hepatic haematomas were smaller than 2 cm, with no clinical or biological effects. Both of these involved ultrasound‐guided marking (*Fig*. 3 *c–f*). The misplacement involved a lesion close to the portal branch of segment III in a patient who had undergone previous right hepatectomy and two metastasectomies from segments II and III. It was not possible to access the new lesion in the remnant of segment III. The fiducial marker was placed 17 mm from the lesion.

At the initial diagnosis, six patients among the study population (14 per cent) presented with peritoneal metastases. Five others developed peritoneal metastases after fiducial placement, with a median time from diagnosis of 18·2 (range 12·2–23·0) months. Of these 11 patients, seven were treated with curative intent with cytoreduction and hyperthermic intraperitoneal chemotherapy. At operation, the location of the peritoneal nodules did not correlate with the needle track. In the other four patients, cross‐sectional imaging showed no sign of perihepatic needle track‐related metastasis.

## Discussion

Fiducial marker placement allowed the elective treatment of all 23 patients with MM, 18 by thermoablation, four with surgery and one with radiotherapy. Two of the four resected patients with MM still had viable malignant cells on pathological analysis. This rate varies widely between 20 and 83 per cent^13^, ^14^, ^15^, ^16^, ^17^, ^18^, ^19^, ^20^, ^21^, ^22^, ^23^, ^24^, with an *in situ* MM recurrence rate reported to occur in 33–74 per cent of lesions^31^, emphasizing the importance of dealing with these lesions in an attempted curative treatment strategy. Strategies to achieve comprehensive hepatic clearance are evolving rapidly, and include PSH^32^, ^33^, which can be combined with ablation to optimize treatment and limit morbidity^34^, ^35^. In retrospective series, the oncological outcomes of these combined strategies are associated with lower progression‐free survival but similar overall survival compared with that achieved by liver resection without ablation^2^, ^34^, ^35^. Although liver‐sparing resection would be the optimal treatment for CRLM after MDT reassessment, thermoablation was adopted for 43 of the 76 marked metastases of the present series, allowing a follow‐up response to chemotherapy.

**Figure 4:**
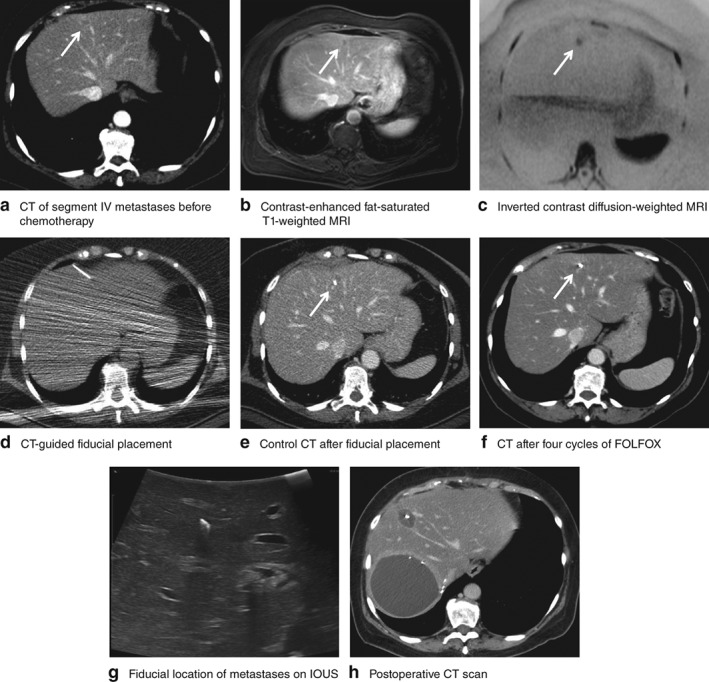
Left colonic adenocarcinoma with four liver metastases in segments IV, VI, VII and VIII. Before chemotherapy, cross‐sectional images from **a** CT, **b** MRI contrast‐enhanced fat‐saturated T1‐weighted image and **c** MRI inverted contrast diffusion‐weighted image showed liver metastasis at risk of being missed (white arrow) in segment IV. After CT‐guided marking (**d**) a control CT scan confirmed good fiducial marker placement (**e**). **f** After four cycles of FOLFOX–bevacizumab, the marked lesion disappeared from the CT scan. **g** The fiducial marker allowed the location of the missing metastasis to be identified easily by intraoperative ultrasonography (IOUS), allowing radiofrequency ablation in addition to a right hepatectomy. **h** A postoperative control CT scan confirmed the good targeting of the ablation

Fiducial placement improved the targeting of small lesions in chemotherapy‐altered liver parenchyma, without impairing further treatments. ‘Difficult to localize’ lesions were those that responded to chemotherapy and almost disappeared. The marker enabled these to be targetable (*Fig*. 4).

Fiducial marker migration is a well recognized phenomenon. For example, a previous study^36^ reported a patient in whom a 2‐mm spherical gold marker dropped from the liver through the vena cava to the hip vein with no adverse reaction after 19 months. Another study^37^ described embolization at the junction of the vena cava and right atrium of a 4‐mm platinum marker implanted for hepatic stereotactic body radiotherapy. The fiducial marker was easily removed with an endovascular procedure, and the patient was asymptomatic. Kitamura and colleagues^38^ studied fiducial migration inside the liver and estimated it to be less than 2·5 mm from the basal position of the marker.

In the present study, a risk factor for migration was the proximity of a lesion to a major hepatic vein. In such cases, the risk may be mitigated by determining the location of regional veins on preimplantation images and planning a safer approach^37^, ^39^.

Misplacement of markers is another clinical issue. This has been estimated previously to affect about 10 per cent of placements, where lesions were located high in the liver (segments VII and VIII) or proximal to major portal pedicles^39^. The fiducial marker misplaced in the present series occurred in a lesion difficult to reach, given previous liver surgery, despite a CT‐guided approach. Hepatic haematomas and fiducial marker migrations were probably due to the proximity to hepatic vessels. These might be avoided by placing the marker at the lesion edge opposite the vessel, by excluding patients on anticoagulant medications or by using a thinner needle with microcoil placement.

Another major concern was the risk of metastatic track seeding. Track dissemination has been well described for hepatocellular carcinoma and is thought to occur in 2·3 per cent of patients^40^, but has not been investigated widely in the field of CRLM^41^, ^42^. In a large analysis of CRLM biopsies performed with 20‐ or 22‐G needles, it was found that 10 per cent had implantation metastases. Accordingly, the authors concluded that these metastases negatively impacted survival in 80 per cent of these patients^43^. In another retrospective study^44^, CRLM‐resected patients who underwent biopsy before referral were compared with those who did not undergo biopsy. Among biopsied patients, 19 per cent had evidence of needle‐track deposits. Operative mortality and morbidity rates in the two groups were similar, but the 4‐year survival rate after liver resection was worse in the group that had a preoperative biopsy: mean(s.e.m.) 32.5(5.5) *versus* 46.7(2.8) per cent (*P* = 0·008)^44^. It has been recommended that biopsies should not be performed in potentially resectable patients^43^, ^44^, ^45^. Despite consistent follow‐up and regular cross‐sectional imaging, there was no evidence of track seeding in the present series. This might reflect the use of effective chemotherapy regimens or technical factors, as the preferred method was to mark the posterior edge of the tumour rather than the tumour itself^25^.

The present study suffers from limitations due to its retrospective analysis. The small number of patients precluded survival analyses. As a result, the impact of fiducial placement strategy on prognosis and *in situ* recurrence remains undetermined. The proportion of patients at risk of MM and the incidence of MM were low, but the management of these patients was challenging, highlighting the importance of a multidisciplinary approach to treat lesions at risk of disappearance.

## Disclosure

The authors declare no conflict of interest.

## Supporting information

Appendix S1 Ethical considerations.
