# Supplementary material for: Evaluation of a strategy using pretherapeutic fiducial marker placement to avoid missing liver metastases
Source: BJS Open. 2019 Feb 22;3(3):344–53. doi: 10.1002/bjs5.50140 (PMC6551408; doi:10.1002/bjs5.50140)
Supplement: Supplementary file 1 — Appendix S1 Ethical considerations. [file BJS5-3-344-s001.docx]

**BJS5_50140**

**Evaluation of a strategy using pretherapeutic fiducial marker placement to avoid missing liver metastases**

**V. Kepenekian, A. Muller, P. J. Valette, P. Rousset, M. Chauvenet, G. Phelip, T. Walter, M. Adham, O. Glehen and G. Passot**

**Appendix S1 Ethical considerations**

When the study was initiated, two types of clinical research were distinguished in France: interventional research, where an intervention was realized specifically for the research, and non-interventional research, where the management of patients did not differ from daily practice. According to French law, the second kind of research did not need to be approved by a research ethics committee as long as the intervention was of low risk and already performed outside the scope of clinical research. However, this research must comply with French national regulation regarding data protection and privacy, according to the specific French laws.

As the marking technique evaluated in this study was already used in certain tertiary centres in France, ethical approval was not required.
